# Supplementary material for: Reduced antigenicity of Omicron lowers host serologic response
Source: bioRxiv. 2022 Feb 15:2022.02.15.480546. Preprint. [Version 1] doi: 10.1101/2022.02.15.480546 (PMC8863144; doi:10.1101/2022.02.15.480546)
Supplement: 1 [file NIHPP2022.02.15.480546V1-supplement-1.pdf]

bioRxiv preprint doi: https://doi.org/10.1101/2022.02.15.480546; this version posted February 15, 2022. The copyright holder for this preprint (which was not certified by peer review) is the author/funder, who has granted bioRxiv a license to display the preprint in perpetuity. It is made available under aCC-BY 4.0 International license.

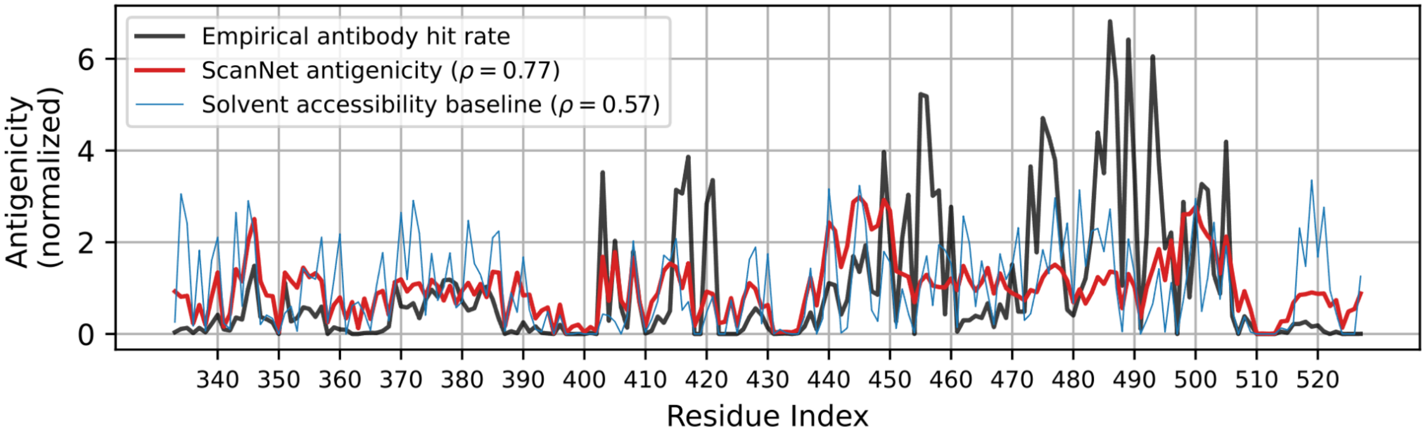

Supplementary Figure S1: Evaluation of overall accuracy of the ScanNet antigenicity profile on the spike protein RBD. ScanNet antigenicity profile for WT RBD (red) vs. the empirical antibody hit rate calculated based on PDB structures (black). RBD residue solvent accessibility is shown as a baseline (blue). The three curves were normalized to the mean value of 1 to facilitate the comparison. The solvent accessibility was computed within the spike trimer in the open state configuration (PDB identifier: 7e5r:A)

bioRxiv preprint doi: https://doi.org/10.1101/2022.02.15.480546; this version posted February 15, 2022. The copyright holder for this preprint (which was not certified by peer review) is the author/funder, who has granted bioRxiv a license to display the preprint in perpetuity. It is made available under aCC-BY 4.0 International license.

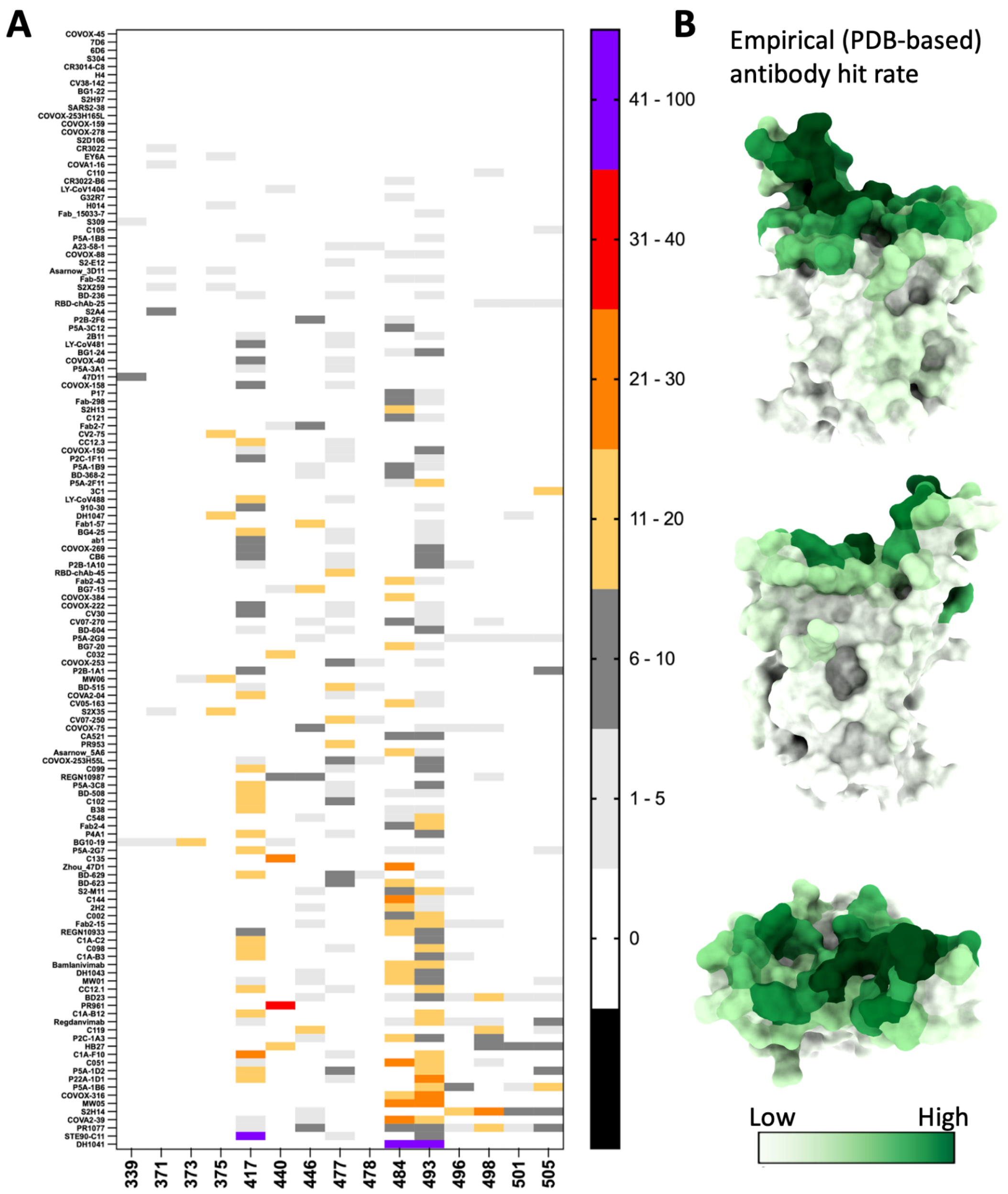

Supplementary Figure S2. Epitope distribution based on solved antibody-RBD structures.
A. Omicron mutations (x-axis) vs. antibody binding. The color-coding corresponds to the fraction of the RBD residue atoms interacting with the antibody. **B.** RBD structure (PDB: 7jvb) is colored according to the antibody hit rate (number of antibodies that interact with the residue).

bioRxiv preprint doi: https://doi.org/10.1101/2022.02.15.480546; this version posted February 15, 2022. The copyright holder for this preprint (which was not certified by peer review) is the author/funder, who has granted bioRxiv a license to display the preprint in perpetuity. It is made available under aCC-BY 4.0 International license.

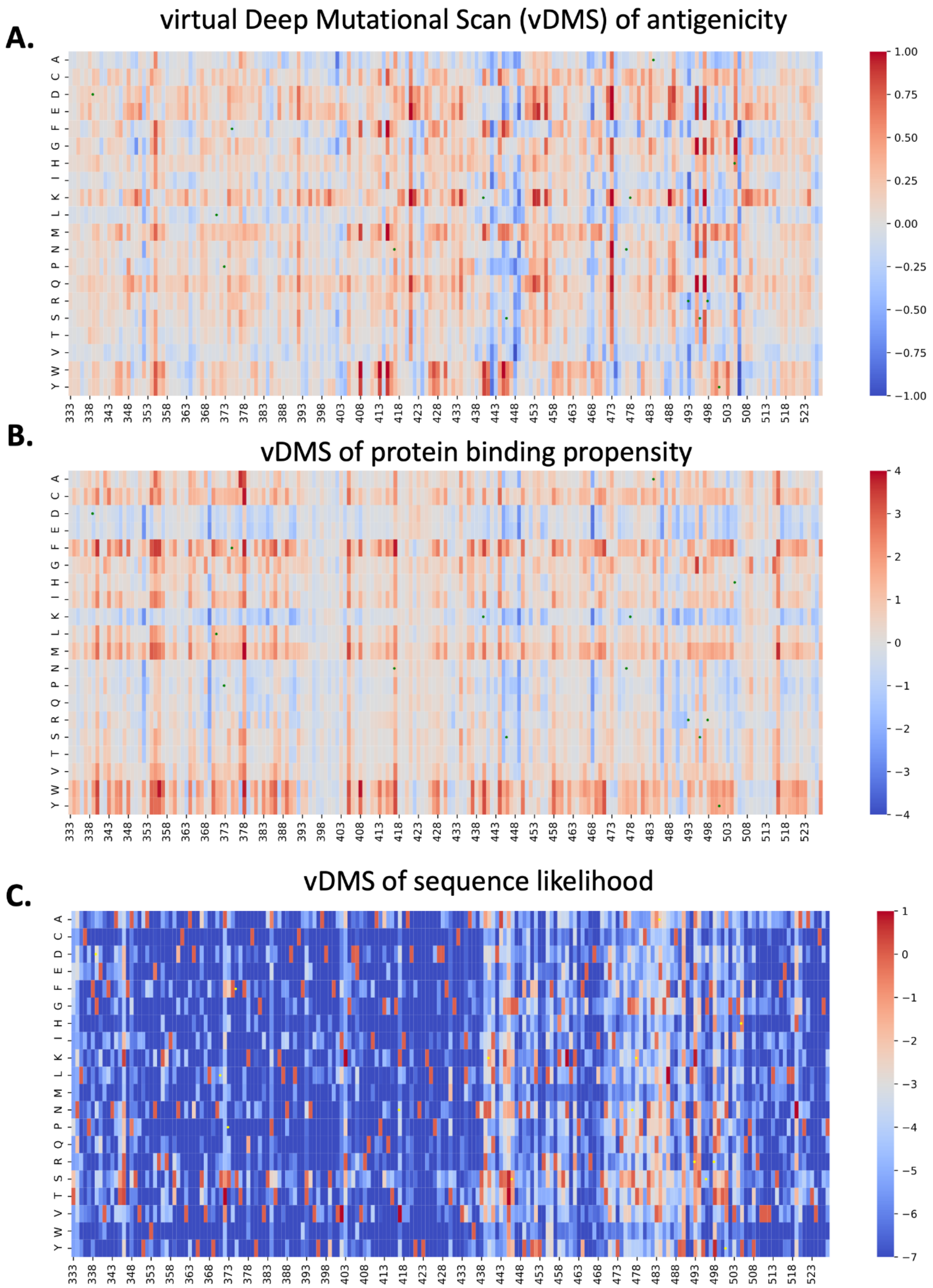

Supplementary Figure S3: virtual Deep Mutational Scans (vDMS) of WT RBD for antigenicity, protein binding propensity and sequence likelihood.

A. vDMS of antigenicity score predicted by ScanNet, see Methods. The difference between the *summed* antigenicity profiles (*over the whole domain*) of mutants and WT is shown. The corresponding distribution of

bioRxiv preprint doi: https://doi.org/10.1101/2022.02.15.480546; this version posted February 15, 2022. The copyright holder for this preprint (which was not certified by peer review) is the author/funder, who has granted bioRxiv a license to display the preprint in perpetuity. It is made available under aCC-BY 4.0 International license.

entries is shown in **Figure 1F, Supplementary Figure S6B**. Green dots indicate Omicron mutations. **B.** vDMS of protein binding propensity score predicted by ScanNet, see Methods. The difference between the *summed* protein binding propensity profiles *(over the whole domain)* of mutants and WT is shown. The corresponding distribution of entries is shown in **Supplementary Figure S6D**. Green dots indicate Omicron mutations. **C.** vDMS of sequence likelihood based on evolutionary records. The difference between the log-likelihood log P(S) of mutant sequence and wild type sequence is shown. The likelihood function was obtained by training a sequence generative model (Restricted Boltzmann Machine), on a multiple sequence alignment of betacoronaviruses RBDs (**Methods**). The vDMS were averaged over five trainings, each using different random seeds. The correlation to the expression level DMS performed in[22] is shown in **Supplementary Figure S7D**. Yellow dots indicate Omicron mutations.

bioRxiv preprint doi: https://doi.org/10.1101/2022.02.15.480546; this version posted February 15, 2022. The copyright holder for this preprint (which was not certified by peer review) is the author/funder, who has granted bioRxiv a license to display the preprint in perpetuity. It is made available under aCC-BY 4.0 International license.

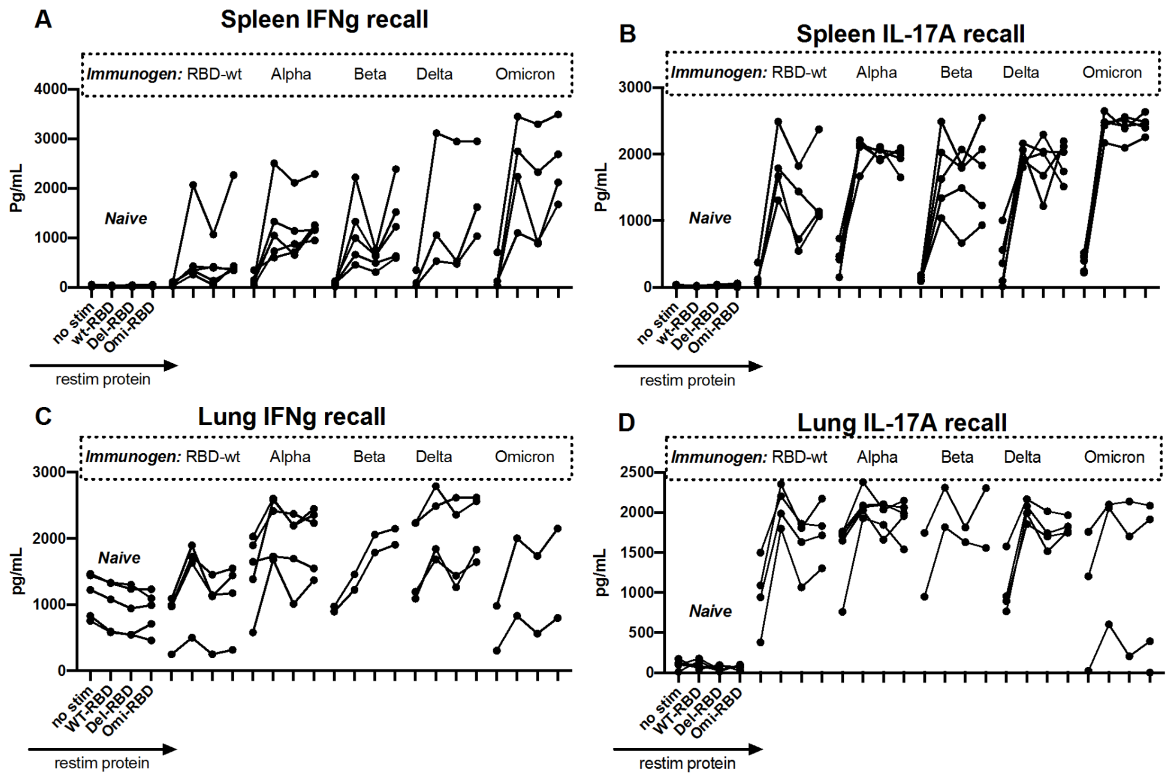

Supplemental Figure S4: The T cell recall responses in the RBD immunized mice. Splenocytes (**A, B**) or lung mononuclear cells (**C, D**) from naïve or RBD immunized mice were left unstimulated or stimulated with WT, Delta, or Omicron RBD for 72h. Culture supernatants were harvested for IFNg and IL-17 measurements by ELISA.

bioRxiv preprint doi: https://doi.org/10.1101/2022.02.15.480546; this version posted February 15, 2022. The copyright holder for this preprint (which was not certified by peer review) is the author/funder, who has granted bioRxiv a license to display the preprint in perpetuity. It is made available under aCC-BY 4.0 International license.

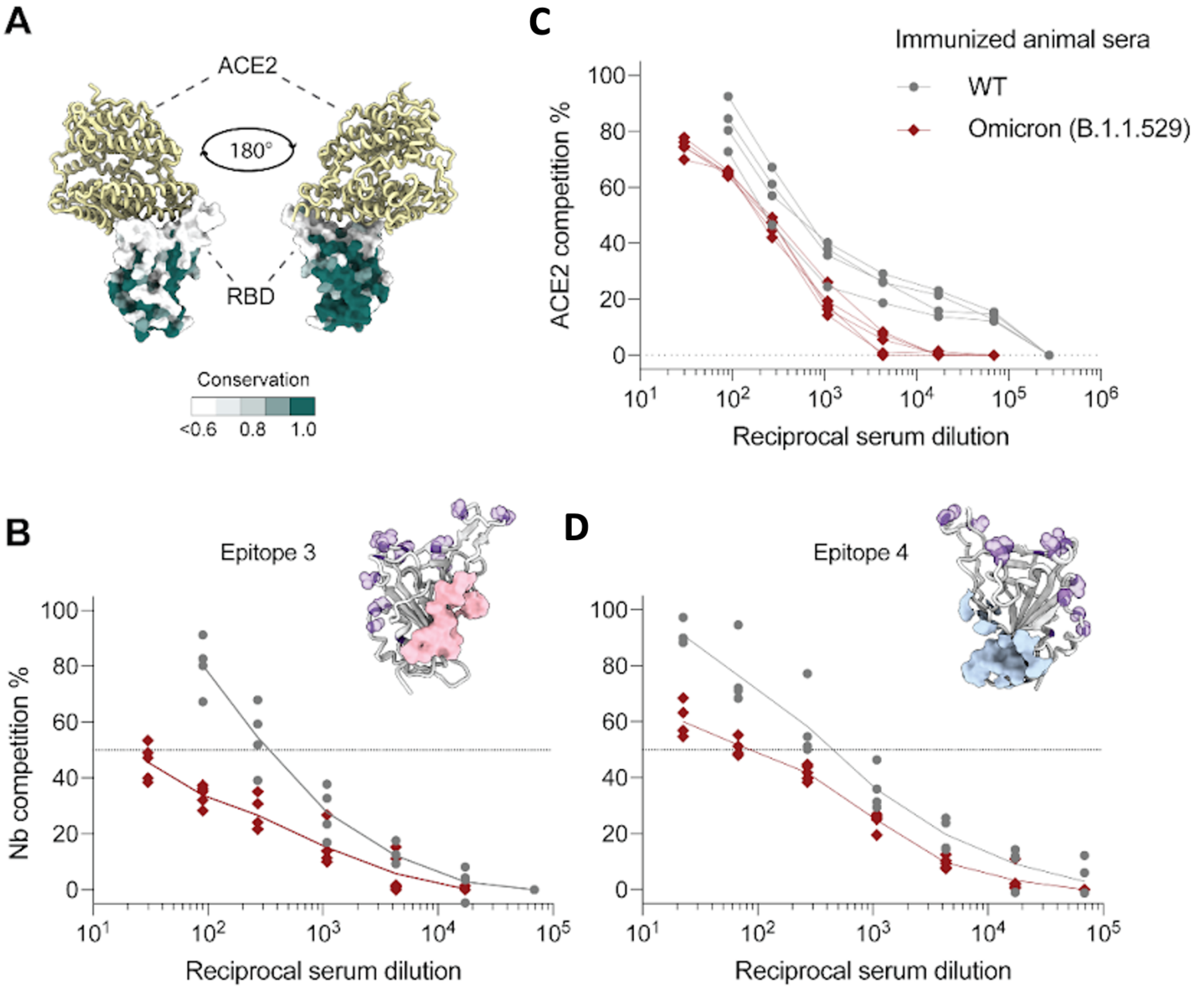

Supplemental Figure S5: Competition ELISA.

A. Structural representations of the RBD-ACE2 complex. The sequence conservation of sarbecovirus RBD was presented in a color gradient, where 1.0 (in dark green) indicates that the residue is 100% conserved within all the sarbecovirus clades. **B-D**. Competitive ELISA between mice sera and (**B**) hACE2, (**C**) a high-affinity nanobody that targets a conserved RBD epitope (residues 337, 351-358, 396, 464, 466-468), or (**D**) a high-affinity nanobody that targets another conserved RBD epitope (residues 380, 381, 386, 390, 393, 428-431, 464, 514-522) for RBD binding. Each plot shows the percentage of remaining ACE2 or Nbs on the immobilized RBD in the presence of sera, expressed as reciprocal serum dilution. RBD was shown as gray ribbons. Mutated residues on Omicron were shown in purple. Distinct, conserved nanobody epitopes (3 and 4) were shown in pink and blue, respectively.

bioRxiv preprint doi: https://doi.org/10.1101/2022.02.15.480546; this version posted February 15, 2022. The copyright holder for this preprint (which was not certified by peer review) is the author/funder, who has granted bioRxiv a license to display the preprint in perpetuity. It is made available under aCC-BY 4.0 International license.

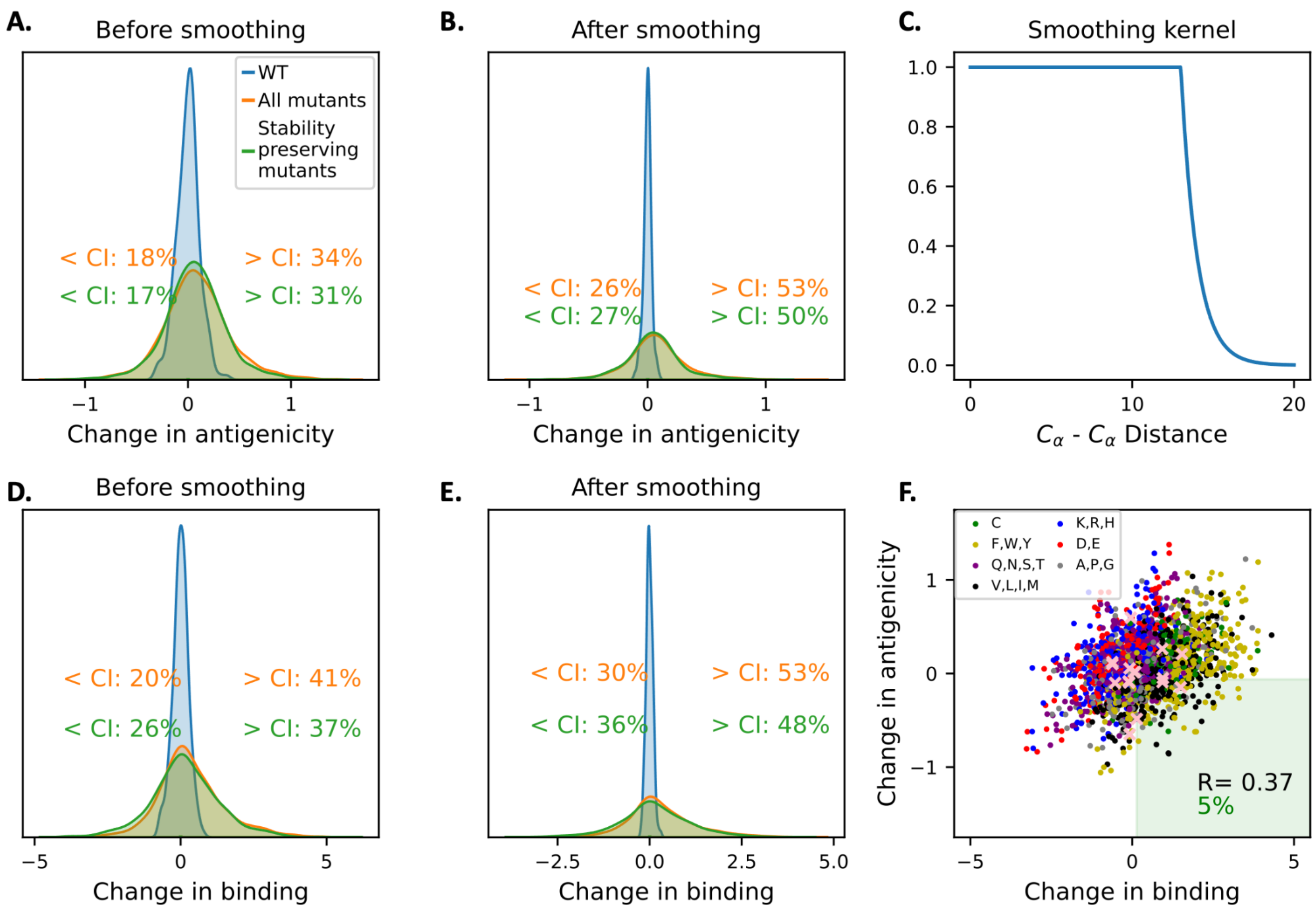

Supplementary Figure S6: Noise estimation and reduction methodology for assessing the impact of all single point mutations on antigenicity and protein binding propensity.

A-B. Histogram of the total change in antigenicity with respect to WT across all single point mutations before (**A**) and after (**B**) smoothing. Blue histogram represents 195 repeated runs of the WT sequence through the comparative modeling pipeline; it corresponds to the noise level induced by homology modeling. Text indicates the fraction of mutations outside of the [5%,95%] confidence interval. **C.** The smoothing kernel used for weighting residues in the neighborhood of the mutation. **D-E.** Histogram of the total change in protein binding propensity with respect to WT across all single point mutations before (D) and after (E) smoothing. **F.** Change in protein binding propensity vs. change in antigenicity for all single-point mutants. Each point corresponds to a single mutation, colored by the type of amino acid (green: cysteine, gold: aromatic, purple: polar, black: hydrophobic, blue: positively charged, red: negatively charged, gray: tiny/proline). The green shaded region denotes mutations that simultaneously increase protein binding propensity and decrease antigenicity; they form a small since both properties are correlated. Pink crosses indicate Omicron mutations.

bioRxiv preprint doi: https://doi.org/10.1101/2022.02.15.480546; this version posted February 15, 2022. The copyright holder for this preprint (which was not certified by peer review) is the author/funder, who has granted bioRxiv a license to display the preprint in perpetuity. It is made available under aCC-BY 4.0 International license.

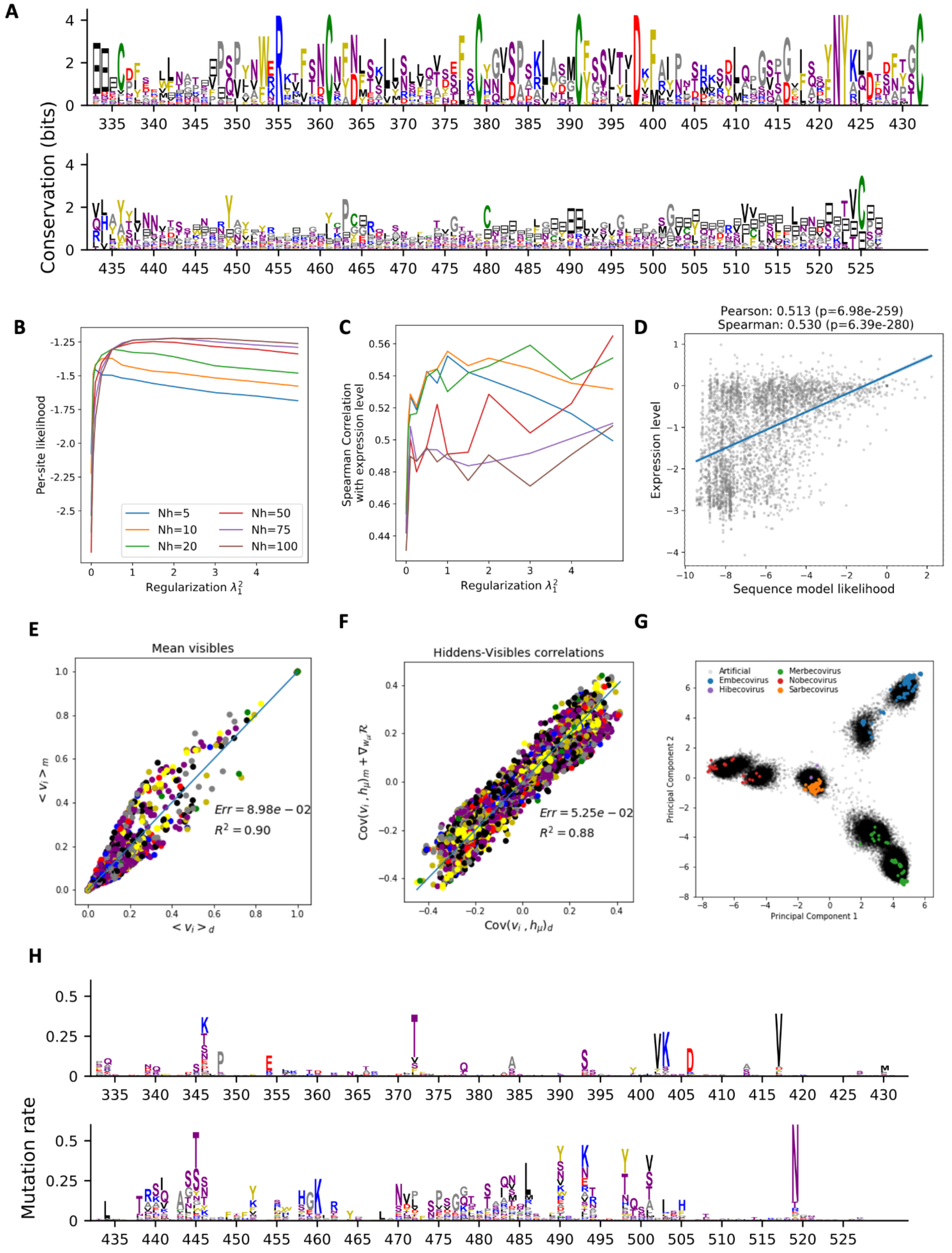

bioRxiv preprint doi: https://doi.org/10.1101/2022.02.15.480546; this version posted February 15, 2022. The copyright holder for this preprint (which was not certified by peer review) is the author/funder, who has granted bioRxiv a license to display the preprint in perpetuity. It is made available under aCC-BY 4.0 International license.

Supplementary Figure S7: **Training, validation and sampling of a sequence generative model for the RBD.**

A. Sequence profile of the MSA of betacoronavirus RBDs identified in UniRef30. **B,C.** Hyperparameter search by cross-validation. (**B**) Cross-validation likelihood (divided by the number of sites) and (**C**) Spearman correlation between the change in sequence log-likelihood and change in expression around WT, as function of the number of hidden units M and of the regularization strength λ_1^2 of the RBM. The pseudo-likelihood (not shown) was also monitored and was highly correlated to the likelihood. Since the three metrics did not peak at the same position, we manually selected $M = 20$, $\lambda_1^2 = 0.5$ as a compromise. **D.** Scatter plot of the change in expression level (experimentally determined in[22]) and the change in log-likelihood for all single point mutants (corresponding matrix shown in **Figure S3C**). **E-G.** Evaluation of the generative properties of the selected model. The distribution of samples generated by the model matched reasonably well the (**E**) single site frequency, (**F**) covariance between visible and hidden units and (**G**) overall topology of the distribution of natural sequences. **H.** Distribution of mutations to WT found in the set of 1000 artificial variants obtained by sampling from $P(S|D_{Hamming}(S, WT) = 15, N_{gaps} = 0)$. For each site, the height of each letter is proportional to the frequency of the corresponding amino acid in the generated set. The total height is proportional to the mutation rate of the site. As expected, RBS is the most variable region.
